# tKeima: A Large-Stokes-Shift Platform for Metal Ion Detection

**DOI:** 10.3390/bios16030178

**Published:** 2026-03-22

**Authors:** Yun Gyo Seo, Dan-Gyeong Han, In Jung Kim

**Affiliations:** 1 Division of Applied Life Sciences, College of Agriculture and Life Science, Gyeongsang National University, Jinju 52828, Republic of Korea; 2 Department of Food Science & Technology, College of Agriculture and Life Science, Gyeongsang National University, Jinju 52828, Republic of Korea

**Keywords:** metal biosensor, fluorescent protein, tKeima, fluorescence quenching, reversibility

## Abstract

Detection of metal ions under complex and heterogeneous conditions is crucial for food safety, environmental monitoring, and cellular studies. Fluorescent proteins (FPs) are attractive biosensors due to their ease of expression, strong emission without external cofactors, and fluorescence quenching upon metal binding. tKeima features a large Stokes shift, pH sensitivity, and spectral stability, reducing background interference and enabling metal detection in complex samples. Here, we examined tKeima quenching toward biologically relevant metal ions (Fe^2+^, Fe^3+^, and Cu^2+^). Metal titration fitted to the Langmuir isotherm yielded dissociation constants (*K*_d_) of 2710.7 ± 178.6 μM (Fe^2+^), 3112.0 ± 176.7 μM (Fe^3+^), and 881.9 ± 76.2 μM (Cu^2+^), with maximum quenching capacities (*B*_max_) of 133.8 ± 2.4%, 128.3 ± 2.5%, and 109.2 ± 1.2%, respectively. Limits of detection were 396.0 μM (Fe^2+^), 428.6 μM (Fe^3+^), and 457.7 μM (Cu^2+^), and linear quenching responses were observed up to ~1000, 1500, and 1000 μM, respectively. Sphere-of-action combined with Stern–Volmer analysis indicated primarily dynamic quenching for Fe^2+^ and Cu^2+^, whereas Fe^3+^ showed a stronger static component. tKeima showed partial fluorescence restoration with ethylenediaminetetraacetic acid and moderate selectivity against interfering ions. These findings clarify tKeima’s metal-quenching mechanism and support its use as a platform for metal-responsive biosensors.

## 1. Introduction

Fluorescent proteins (FPs) have revolutionized biological imaging by enabling real-time, noninvasive, and highly sensitive visualization of cellular and molecular processes. Their ease of expression in living cells and ability to emit fluorescence signals without the need for external cofactors make them indispensable tools in modern cellular biology. These properties not only facilitate direct detection using standard fluorescence microscopy or spectroscopy but also position FPs as promising components in biosensor development, particularly for monitoring dynamic environmental changes within biological systems [1].

Fluorescence quenching upon binding to specific metal ions offers a valuable mechanism for designing metal ion-sensitive biosensing platforms [2]. This is particularly relevant in applications such as cellular process investigation, environmental monitoring, biomedical diagnostics, food safety assessment, and industrial process control, where accurate and timely detection of metal ions is critical. FP-based metal biosensors provide distinct advantages, including real-time operation, compatibility with live-cell imaging, and high spatial resolution without the requirement for exogenous cofactors [3]. The sensing principle primarily relies on fluorescence quenching induced by specific interactions between the metal ion and the chromophore or its immediate microenvironment [4]. Such interactions may involve direct coordination to the chromophore’s π-electron system, binding to nearby amino acid residues (e.g., histidine, cysteine, glutamate, aspartate), or perturbations of the local electrostatic and hydrogen-bonding network, ultimately altering the excited-state properties of the chromophore [5,6]. These molecular processes underpin the selectivity and sensitivity achievable in FP-based metal ion biosensing platforms. Previously, various FPs have been proven to act as effective metal biosensors. For example, mNeonGreen has been engineered to selectively detect Hg^2+^ through metal-induced fluorescence quenching [7] while flavin-based FP variants have been developed for the detection of Zn^2+^ and Cu^2+^ by exploiting changes in chromophore fluorescence upon metal coordination [8]. While many FP-based metal sensors are designed for high sensitivity, rapid fluorescence recovery, and high selectivity, these traits can be problematic in complex biological or environmental samples [9,10]. Excessive sensitivity may cause over-quenching at normal metal levels, leading to unstable signals, while narrow selectivity or rapid reversibility can complicate measurements in heterogeneous matrices. Therefore, there is also a need for FP-based metal sensors that balance sensitivity, reversibility, and selectivity while maintaining robust and stable signals in complex conditions. 

Keima proteins, first identified from *Montipora* coral, are characterized by an exceptionally large Stokes shift and high pH sensitivity, which together minimize background autofluorescence and enhance detection accuracy in complex environments [11]. Several engineered variants, including mKeima, dKeima, and tKeima, have subsequently been developed [11]. tKeima, an orange-emitting FP from the Keima family, is distinguished by its high pH sensitivity, spectral stability, and large Stokes shift [12]. It exhibits excitation and emission maxima at 440 nm and 617 nm, respectively, under acidic conditions [12]. As a tetramer composed of four covalently linked Keima units, tKeima is the ancestor of its dimeric and monomeric counterparts, such as dKeima and mKeima [11].

Despite the widespread use of FPs as metal-responsive biosensors, the fluorescence behavior of tKeima in the presence of transition metals has not been systematically explored. In particular, its quenching response and reversibility toward biologically relevant metal ions remain unknown. This knowledge gap is critical, as understanding how tKeima interacts with specific ions could expand the functional versatility of the Keima family beyond pH sensing. In this study, we provide the first comprehensive evaluation of tKeima’s fluorescence response to metal ions such as Fe^2+^, Fe^3+^, and Cu^2+^. By quantifying binding affinity and quenching capacity, and by probing the underlying mechanisms of fluorescence modulation, we aimed to establish tKeima as a promising platform for designing FP-based metal ion detection. We further evaluated its practical applicability as a metal biosensor by assessing sensing performance in terms of limit of detection (LOD), limit of quantification (LOQ), reversibility, and selectivity. This study will help expand the application scope of the Keima family by providing the first evaluation of tKeima’s fluorescence response to transition metal ions and by demonstrating practical insights into metal detection under complex and heterogeneous environments.

## 2. Materials and Methods

### 2.1. Protein Preparation

Gene cloning, as well as protein expression and purification for tKeima, were performed as previously reported [13]. Briefly, the tKeima gene was expressed in *Escherichia coli* BL21 (DE3) cells and purified by nickel–nitrilotriacetic acid affinity chromatography followed by size-exclusion chromatography.

### 2.2. Spectroscopic Analysis

Purified tKeima was characterized by spectral scanning to determine its maximum excitation and emission wavelengths, measured within the ranges of 350–650 nm and 480–700 nm, respectively. To assess the fluorescence response of tKeima to metal ions, the protein–metal mixtures were excited at 450 nm, and fluorescence emission was monitored at 617 nm. All samples were loaded into a 96-well black microplate and gently mixed in an orbital shaker for 10 s prior to measurement. Fluorescence readings were obtained using a Synergy H1 microplate reader (BioTek Instruments, Inc., Winooski, VT, USA). All measurements were conducted in triplicate at 25 °C. Correction for inner-filter effects was considered unnecessary due to their negligible influence under the tested conditions. The detector gain was fixed at 120, with excitation and emission bandwidths of 9 nm and 15 nm, respectively. Fluorescence signals were collected using top-read mode with an integration time of 100 ms.

### 2.3. Metal Screening

Metal ion screening was conducted using purified tKeima at micromolar concentrations, prepared in Tris–HCl buffer (pH 8.0), and 10 mM solutions of various metal chlorides. FeCl_2_, FeCl_3_, and CuCl_2_ (≥97%, Sigma-Aldrich, St. Louis, MO, USA) were used as the primary quenching ions, while LiCl, NaCl, MgCl_2_, CaCl_2_, MnCl_2_, CoCl_2_, and NiCl_2_ at analytical grade were obtained from TCI (Tokyo, Japan) or Daejung (Siheung, South Korea). For visual inspection of fluorescence quenching, 50 µL of the tKeima solution was mixed with 50 µL of each metal ion solution in distilled water and incubated at 25 °C for 5 min. The mixtures were then illuminated with an LED transilluminator (KA-33-61, Korea Ace Scientific, Seoul, South Korea). For quantitative analysis, fluorescence emission of identical mixtures was measured as described above. Relative fluorescence quenching (%) represents the fluorescence intensity of tKeima in the presence of metal ions relative to that in the absence of metal ions (control).

### 2.4. Quenchable Metal Titration

Titration experiments with Fe^2+^, Fe^3+^, and Cu^2+^ were performed. A 4 µM solution of purified tKeima (50 µL) was mixed with an equal volume of FeCl_2_, FeCl_3_, or CuCl_2_ at concentrations ranging from 0 to 20,000 µM and incubated at 25 °C for 5 min. To determine the dissociation constant (*K*_d_) and maximum binding capacity (*B*_max_) of tKeima for these metal ions, titration data for Fe^2+^, Fe^3+^, and Cu^2+^ were fitted to a Langmuir isotherm using SigmaPlot 15.0 software (Systat Software, Erkrath, Germany). The Langmuir isotherm used for data fitting is presented below [14]:

$$\mathrm{Relative}\;\mathrm{fluorescence}\;\mathrm{quenching}\;(\%)\;=\;\frac{B_{max}\;{\left[M\right]}_{unbound}}{K_{d}+{\left[M\right]}_{unbound}}$$

*K*_d_ represents the equilibrium dissociation constant (µM), and *B*_max_ denotes the maximum binding capacity or the concentration of available binding sites. Since the amount of metal ions bound to tKeima was represented by relative fluorescence quenching (%) in this study, *B*_max_ is defined as the maximum fluorescence quenching observed. [M]_unbound_ corresponds to the equilibrium concentration of metal ions that remain unbound to the protein (µM). When the total metal ion concentration ([M]_total_) is in large molar excess relative to that of tKeima, [M]_unbound_ can be assumed to be equivalent to [M]_total_.

### 2.5. Quenching Mechanism

To investigate the quenching mechanism, Stern–Volmer analysis was conducted by measuring the fluorescence intensities of tKeima in the presence of various concentrations of Fe^2+^, Fe^3+^, or Cu^2+^ at two temperatures: 25 °C and 35 °C [14]. The Stern–Volmer plots were generated by plotting *F*_o_/*F* against the metal ion concentration [Q]. The relative fluorescence quenching (*F*_o_/*F*) was determined by dividing the fluorescence intensity of metal-free tKeima (*F*_o_) by the intensity in the presence of metal ions (*F*). The Stern–Volmer constant (*K*_SV_) was obtained from the slope of the linear regression line:

$$\frac{Fo}{F}=(1+K_{sv}[Q])$$

The quenching mechanism was also examined based on the sphere-of-action model [15,16]. The dynamic (*K*_D_) and static (*K*_S_) quenching constants were obtained by fitting the experimental data to the following sphere-of-action equation:

$$\frac{Fo}{F}=(1+K_{D}[Q])e^{k_{s}[Q]}$$

where *K*_D_ and *K*_S_ represent the dynamic and static quenching constants (M^−1^), respectively, and [Q] is the concentration of metal ions. The fitting of quenching data was performed using nonlinear regression analysis to determine the best-fit parameters and the coefficient of determination (*R*^2^).

### 2.6. Reversibility and Selectivity

To assess the reversibility of metal-induced fluorescence quenching of tKeima, quenched samples were prepared by mixing 50 µL of 4 µM tKeima with 50 µL of 20 mM Fe^2+^, Fe^3+^, or Cu^2+^ solutions, followed by incubation at 25 °C for 5 min. The quenched state of each mixture was confirmed by measuring fluorescence emission. Subsequently, 50 µL of ethylenediaminetetraacetic acid (EDTA) solution (at final concentrations of 1, 5, 10, 50, 100, and 500 mM) was added to each quenched mixture. After incubation at 25 °C for 30 min, fluorescence emission was measured to evaluate recovery.

To evaluate the selectivity of tKeima toward Fe^2+^, Fe^3+^, and Cu^2+^, 50 µL of 4 µM tKeima was mixed with 25 µL of 2 mM Fe^2+^, Fe^3+^, or Cu^2+^ solutions and 25 µL of 2 mM solutions of potentially interfering various metal ions. As a control, mixtures containing only Fe^2+^, Fe^3+^, or Cu^2+^ without additional metal ions were prepared. All mixtures were incubated at 25 °C for 5 min and subsequently subjected to fluorescence measurement.

### 2.7. Structure Analysis and Metal Ion Docking

The tetrameric interface of tKeima was analyzed using PDBePISA [17]. Sequence alignment of tKeima with Dronpa and iq-mEmerald was performed using Clustal Omega [18] and visualized with ESPript 3.0 [19]. Cu^2+^ and Fe^3+^ were docked to tKeima using AlphaFold3 (AF3) (https://alphafoldserver.com/) [20]. Structural figures were visualized using PyMOL (https://www.pymol.org/ (accessed on 6 October 2025)).

### 2.8. Whole-Cell Fluorescence Quenching Assay

*E. coli* BL21 (DE3) cells expressing tKeima were prepared as IPTG-induced (+ IPTG, 0.5 mM isopropyl β-D-1-thiogalactopyranoside) and non-induced (− IPTG) groups to assess whole-cell applicability in a cellular matrix and to distinguish tKeima-dependent signals. For this purpose, the cells were cultivated, harvested, washed, and resuspended in a buffer composed of a potassium phosphate (200 mM, pH 7.4) supplemented with NaCl (50 mM). An equal wet cell mass (0.2 g) from each group was resuspended, and the cell density was adjusted to the same optical density at 600 nm (OD_600_). Normalized suspensions were incubated with metal ions at the indicated concentrations for 5 min at 25 °C, and fluorescence was measured in a 96-well black plate. Relative fluorescence quenching (%) was calculated by comparing fluorescence intensities with and without metal ions.

## 3. Results and Discussion

### 3.1. Metal Ion-Induced Fluorescence Quenching of tKeima: Pronounced Responses to Fe^2+^, Fe^3+^, and Cu^2+^

**Figure 1:**
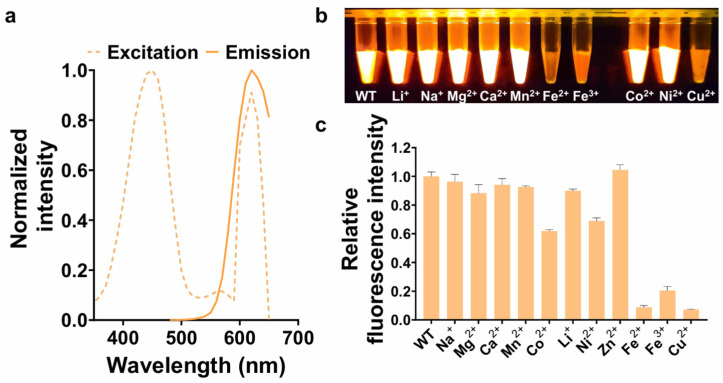
Metal-induced fluorescence quenching of tKeima. (**a**) Spectral characterization of purified tKeima. (**b**) Representative images showing metal ion-induced fluorescence quenching of tKeima. Equal volumes (50 µL) of 4 µM tKeima and 10 mM metal ion solutions were mixed and exposed to LED illumination. (**c**) Relative fluorescence intensities of tKeima in the presence of various metal ions. WT, wild type.

To validate the purified tKeima protein, the maximum excitation and emission wavelengths were measured using spectral scanning (Figure 1a). The purified tKeima exhibited fluorescence with excitation and emission maxima at approximately 450 nm and 617 nm, respectively. These values are consistent with previously reported spectral properties of tKeima [13].

To assess the fluorescence quenching response of tKeima, we tested a range of metal ion solutions, including Na^+^, Mg^2+^, Ca^2+^, Mn^2+^, Co^2+^, Li^+^, Ni^2+^, Zn^2+^, Fe^2+^, Fe^3+^, and Cu^2+^, as a standard panel commonly used in fluorescent protein–based metal-sensing systems [2], each of which was individually incubated with the protein. Toxic heavy metals, such as Cd^2+^, Hg^2+^, and Pb^2+^, belonging to a distinct category of non-essential and highly toxic metals, were not included at this stage. The mixture of tKeima and each metal ion was initially visualized under LED illumination to assess changes in fluorescence intensity (Figure 1b). As a result, significant reductions in fluorescence were observed in the tKeima samples treated with Fe^2+^, Fe^3+^, and Cu^2+^. These changes were quantitatively assessed by measuring fluorescence emission at 617 nm (Figure 1c). Compared with the control without metal ion incubation, fluorescence emission of tKeima was significantly reduced by 91.28%, 79.51%, and 92.87% upon treatment with Fe^2+^, Fe^3+^, and Cu^2+^, respectively. Co^2+^ and Ni^2+^ also reduced the fluorescence intensity by 38.16% and 31.1%, respectively. Na^+^, Mg^2+^, Ca^2+^, Mn^2+^, Li^+^, and Zn^2+^ induced modest reductions in fluorescence intensity, with decreases of 3.68%, 11.76%, 5.97%, 7.45%, 10.03%, and 0%, respectively. Therefore, Fe^2+^, Fe^3+^, and Cu^2+^, which exhibited pronounced fluorescence quenching in the initial screening and have biological and practical relevance, were selected for the subsequent experiments.

### 3.2. Titration of tKeima with Fluorescence-Quenchable Metal Ions

**Figure 2:**
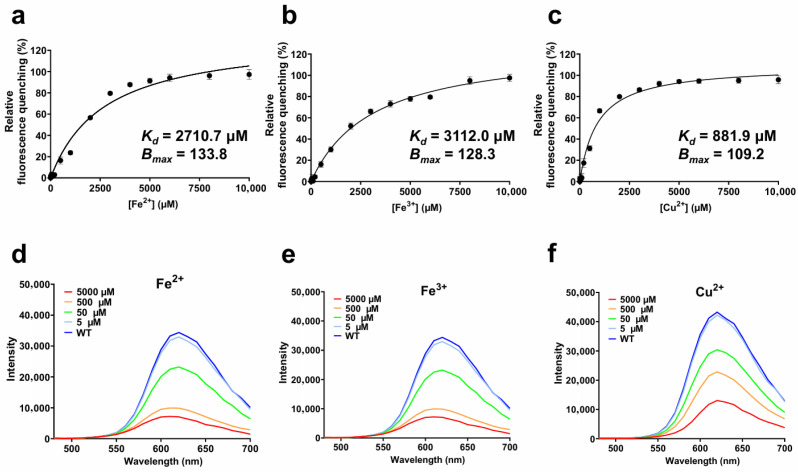
Metal titration of tKeima and fluorescence emission spectra in the presence of each metal ion. (**a**–**c**) tKeima was titrated with various concentrations of Fe^2+^, Fe^3+^, and Cu^2+^ (0 to 10,000 µM at final concentrations). The data fitting was performed using the one metal-binding mode with the Langmuir equation in SigmaPlot. Data represents standard deviations of three replicates. (**d**–**f**) The fluorescence spectra of tKeima in the presence of Fe^2+^, Fe^3+^, or Cu^2+^. *K*_d_, the equilibrium dissociation constant (µM); *B*_max_, the maximum binding capacity.

To perform titration assays toward Fe^2+^, Fe^3+^, and Cu^2+^, which exhibited considerable fluorescence quenching in the prior results (Figure 1), the protein was exposed to a range of metal ion concentrations (Figure 2a). Titration of tKeima with Fe^2+^ resulted in fluorescence reductions of 3.34%, 16.38%, 23.69% and 91.33% at 50, 500, 1000, and 5000 µM, respectively (Figure 2b). In the case of Fe^3+^, the fluorescence intensity decreased by 3.12%, 16.19%, 30.05%, and 77.82% at the same concentrations, respectively. Cu^2+^ induced the strongest quenching effect, with reductions of 1.43%, 31.32%, 66.62%, and 94.19%, respectively, indicating its higher potency even at low concentrations.

To assess the binding characteristics of each metal ion, fluorescence quenching data were fitted to the Langmuir isotherm model, which assumes a 1:1 reversible binding interaction between metal ion and the protein [14]. The fitting yielded *K*_d_ values of 2710.7 ± 178.6 µM for Fe^2+^, 3112.0 ± 176.7 µM for Fe^3+^, and 881.9 ± 76.2 µM for Cu^2+^, alongside corresponding *B*_max_ values of 133.8 ± 2.4, 128.3 ± 2.5, and 109.2 ± 1.2, respectively (Figure 2a)*;* all fits showed high goodness of fit (*R*^2^ > 0.95). These results demonstrate that although Cu^2+^ has the highest binding affinity (lowest *K*_d_), Fe^2+^ and Fe^3+^ show the greatest maximum quenching capacity (*B*_max_). Taken together, the apparent binding efficiency followed the order of Cu^2+^ > Fe^2+^ ≈ Fe^3+^ in terms of affinity and Fe^2+^ > Fe^3+^ > Cu^2+^ in terms of capacity. The *B*_max_ values higher than 100% in the cases of Fe^2+^ and Fe^3+^ might be explained by the oligomeric nature of tKeima and the presence of multiple accessible binding residues on its β-barrel surface. Given that tKeima forms a tetramer, the effective number of metal-binding events, particularly for Fe^2+^ and Fe^3+^ per protein complex, may exceed one, thereby leading to an apparent overestimation of *B*_max_ in the fitting model.

To evaluate how pH influences metal binding, additional titrations were performed for Fe^2+^, Fe^3+^, and Cu^2+^ at pH 5.0, 7.4, 8.0, and 9.0 (Supplementary Figure S1). Although the baseline fluorescence intensity of tKeima decreased under mildly acidic conditions (pH 5.0), the fitted *K*_d_ values were similar across the tested pH values (Fe^2+^: 3042–3898 µM; Fe^3+^: 3440–4073 µM; Cu^2+^: 739.2–846.1 µM). These observations indicate that, within the physiologically and environmentally relevant pH range of 5–9, tKeima retains comparable affinity for metal ions and that its metal-induced quenching behavior is primarily governed by metal concentration rather than pH.

When compared with other FPs, tKeima shows a moderate response to metal ions. In previous studies, DendFP exhibited strong fluorescence quenching even at tens of micromolar concentrations of Fe^2+^, Fe^3+^, and Cu^2+^, with *K*_d_ values of 24.6, 41.7, and 137.2 µM, respectively [14], and Dronpa showed a sharp decrease in fluorescence intensity at around 5–10 µM Cu^2+^ [21]. In contrast, tKeima and ZsYellow required much higher metal concentrations, typically several hundred micromolar, to induce comparable fluorescence changes, responding in a more gradual and concentration-dependent manner [22]. Overall, compared with these highly responsive FPs, tKeima exhibits lower sensitivity towards metal ions.

Although highly sensitive FPs are capable of detecting trace-level metal ions sensitively, as required for regulatory-level environmental monitoring, their over-responsiveness can pose challenges in real-world metal-sensing applications, which involve moderate to high metal concentrations. For example, even a physiologically normal intracellular system already contains basal levels of metals, which can cause background interference in highly sensitive biosensors [23]. In environmental samples, such as industrial and agricultural wastewaters, in which metal ions are typically present at high levels, sensitive biosensors may cause excessive quenching, leading to rapid signal saturation or pronounced signal fluctuations even in response to small concentration changes. These effects can complicate data interpretation, reduce reproducibility and the quantifiable dynamic range, and increase background interference [24]. Conversely, tKeima’s moderate sensitivity effectively compensates for these limitations associated with over-responsive metal biosensors. It provides a practical advantage for applications involving higher metal ion concentrations by offering robust and stable fluorescence readout over a broader and higher concentration range [25]. It is appropriate for first-line on-site assessment, where speed, operational simplicity, and minimal instrumentation are prioritized. Furthermore, its large Stokes shift minimizes spectral overlap and self-absorption, reducing interference from cellular autofluorescence, dissolved organic matter, and other background signals commonly present in environmental samples [26]. Together, these properties enable tKeima to reliably reflect metal-dependent fluorescence changes across diverse matrices, from complex cellular systems to heterogeneous environmental samples, enhancing the clarity and interpretability of fluorescence-based metal sensing experiments. However, to compensate for its relatively low sensitivity, in future applications, tKeima could be coupled with established analytical workflow sample pre-concentration, metal enrichment, or external signal-amplification modules to extend the effective detection limit [27].

To evaluate whether this quenching was accompanied by structural rearrangements of the protein—partial unfolding or changes in the spatial organization of the β-barrel—or by alterations in the chromophore environment, including changes in solvent exposure, local polarity, or hydrogen bonding, emission spectra were recorded at each titration point (Figure 2b). The spectra revealed no detectable shifts in emission maxima under any tested condition, indicating that the quenching process likely occurs without major structural perturbation in the chromophore environment of tKeima.

### 3.3. Quenching Mechanism

**Figure 3:**
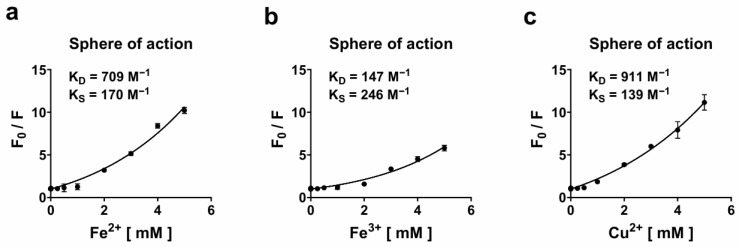
Sphere-of-action analysis of the tKeima quenching system by (**a**) Fe^2+^, (**b**) Fe^3+^, and (**c**) Cu^2+^ at 25 °C. The plots represent nonlinear fitting of the fluorescence quenching data according to the sphere-of-action equation. The values of *K*_D_ and *K*_S_ indicate the dynamic and static quenching constants (M^−1^), respectively, and [Q] represents the concentration of metal ions. *F*_o_ and *F* correspond to the fluorescence intensities of tKeima in the absence and presence of metal ions, respectively. All measurements were performed at 25 °C, and the data are expressed as means ± standard deviations from three independent experiments.

To investigate the mechanism of quenching—static or dynamic—Stern–Volmer analysis was performed based on the relative fluorescence intensities (*F*_o_/*F*) at two temperatures: 25 °C and 35 °C (Supplementary Figure S2). The distinction between dynamic and static quenching mechanisms lies in their underlying molecular interactions [28]. Dynamic quenching occurs through collisional encounters between the fluorophore and quencher during the excited state and is typically enhanced at higher temperatures due to increased molecular diffusion. Conversely, static quenching involves the formation of a non-fluorescent ground-state complex between the fluorophore and quencher, which often exhibits negligible or even reduced quenching efficiency with increasing temperature due to complex dissociation. For both Fe^2+^ and Cu^2+^, a higher *K*_sv_, represented by the slope, was observed at 35 °C compared to that observed at 25 °C, indicating increased quenching efficiency at elevated temperature and supporting a dynamic (collisional) quenching mechanism. In contrast, Fe^3+^ displayed negligible temperature dependence in its *K*_sv_ values, suggesting a distinct or less temperature-sensitive interaction mode, possibly involving weaker or partially static components. Although showing a clear temperature-dependent trend in fluorescence quenching, the Stern–Volmer analysis did not exhibit reliable linearity, suggesting a distinct underlying mechanism for tKeima. It was therefore hypothesized that tKeima may undergo a mixed quenching mechanism. To examine this, fluorescence quenching data at 25 °C were fitted to the sphere-of-action model [15,16] (Figure 3); all fits showed high goodness-of-fit (*R*^2^ > 0.98), which accounts for both dynamic and static quenching processes and enables the simultaneous determination of two parameters: the dynamic quenching constant (*K*_D_) and the static quenching constant (*K*_S_). The nonlinear fitting of the fluorescence quenching data showed excellent correlation with the experimental results, confirming that the sphere-of-action model accurately describes the metal-induced quenching behavior of tKeima. The fitted parameters yielded *K*_D_ and *K*_s_ values of 0.000709 ± 0.000072 × 10^6^ and 0.000170 ± 0.000017 × 10^6^ L mol^−1^ for Fe^2+^ (*R*^2^ = 0.9865), 0.000147 ± 0.000020 × 10^6^ and 0.000246 ± 0.000017 × 10^6^ L mol^−1^ for Fe^3+^ (*R*^2^ = 0.9973), and 0.000911 ± 0.000018 × 10^6^ and 0.000139 ± 0.000003 × 10^6^ L mol^−1^ for Cu^2+^ (*R*^2^ = 0.9961), respectively. When comparing these constants, Fe^2+^ exhibited a *K*_D_ value approximately 4.8 times higher than that of Fe^3+^, while Cu^2+^ showed the highest dynamic quenching constant, about 6.2 times greater than that of Fe^3+^. This indicates that Fe^2+^ and Cu^2+^ induce more efficient dynamic quenching through frequent diffusional encounters with the fluorophore. In contrast, Fe^3+^ exhibited a *K*_S_ value about 1.4 times higher than that of Fe^2+^ and 1.8 times higher than that of Cu^2+^, indicating a greater static or sphere-of-action contribution. This suggests that Fe^3+^ ions might be electrostatically attracted to negatively charged residues on the protein surface, where they may form non-emissive complexes or remain in close proximity to the chromophore, thereby quenching fluorescence without direct collisions. Mechanistically, the larger *K*_D_ values of Fe^2+^ and Cu^2+^ indicate that their quenching mainly arises from rapid diffusional interactions with the excited chromophore, consistent with a dynamic process. In contrast, the relatively higher *K*_S_ of Fe^3+^ suggests the formation of localized, non-emissive interactions near the chromophore, implying a partial static contribution. Overall, these results demonstrate that the quenching behavior of tKeima is governed by a mixed mechanism, where the relative contributions of dynamic and static processes vary according to the metal ion’s charge and coordination properties. In combination with the temperature-dependent Stern–Volmer analysis, these results further verify that tKeima follows a mixed quenching mechanism. The mixed quenching property might also be explained by its tetrameric architecture, which inherently creates heterogeneous metal accessibility for the chromophores in different subunits.

### 3.4. Reversibility of Metal-Induced Quenching

**Figure 4:**
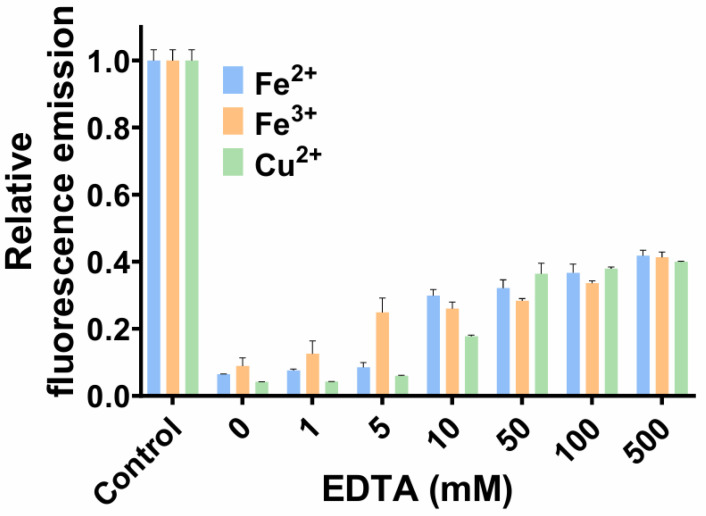
Reversibility of tKeima fluorescence quenching. Various concentrations of EDTA were added (as chelators) to the tKeima samples quenched using Fe^2+^, Fe^3+^, and Cu^2+^. Data represent the means ± standard deviations of three replicates. EDTA, ethylenediaminetetraacetic acid.

The reversibility of fluorescence quenching is a critical parameter that determines the reusability of FP-based biosensors [29]. In this study, EDTA was employed as a competitive chelator to sequester metal ions from the protein–metal complexes. The quenched state of tKeima was prepared by mixing 50 µL of 4 µM tKeima with an equal volume of 20 mM Fe^2+^, Fe^3+^, or Cu^2+^ solution, followed by incubation for 5 min at 25 °C. Subsequently, EDTA was added to yield final concentrations of 1, 5, 10, 50, 100, and 500 mM (Figure 4). In the case of Fe^2+^, fluorescence recovery, defined as the relative increase in fluorescence intensity after EDTA addition normalized to the difference between the control and quenched states, increased progressively with various concentrations of EDTA, reaching 6.4%, 7.5%, 8.5%, 29.8%, 32.1%, 36.7%, and 41.8% for 1, 5, 10, 50, 100, and 500 mM EDTA, respectively. Fe^3+^-quenched samples exhibited recoveries of 8.8%, 12.5%, 24.8%, 25.9%, 28.3%, 33.6%, and 41.35% under the same conditions. Cu^2+^-quenched samples showed relatively lower recovery at low EDTA concentrations (4.1%, 4.2%, and 5.9% at 1, 5, and 10 mM, respectively), but a rapid increase from 50 mM EDTA, reaching 17.7%, 36.4%, 37.8%, and 39.9% at 50, 100, and 500 mM EDTA. For all three metal ions, there was no distinctive increase in fluorescence recovery above 50 mM EDTA, indicating that the recovery of metal-quenched fluorescence reached saturation. Fluorescence recovery of all three metal-quenched samples nearly reached saturation at 50 mM EDTA, as no further increase was observed at higher concentrations.

The reversibility of quenching observed with EDTA treatment in this study supports the interpretation that quenching occurs through reversible coordination at surface-exposed residues rather than irreversible chromophore damage [14]. The partial recovery observed even at high EDTA concentrations suggests that a fraction of the quenching is irreversible under the tested conditions. This irreversibility may arise from slow dissociation kinetics of tightly bound metal ions or from minor conformational rearrangements in the protein that persist after metal removal. In the case of Cu^2+^, the lower recovery at low EDTA levels is consistent with its higher binding affinity (lowest *K*_d_ in Langmuir analysis) compared with Fe^2+^ and Fe^3+^, requiring a greater chelator excess to effectively disrupt the complex. Mechanistically, EDTA competes with the potential metal-binding residues of tKeima by forming highly stable metal–EDTA complexes [30]. The efficiency of recovery thus reflects both the thermodynamic stability and kinetic lability of the protein–metal complex. Comparable partial reversibility has been reported in other FPs and biosensors, where high-affinity or multi-site metal binding can lead to incomplete restoration of fluorescence even after chelation [14,29].

### 3.5. Selectivity

**Figure 5:**
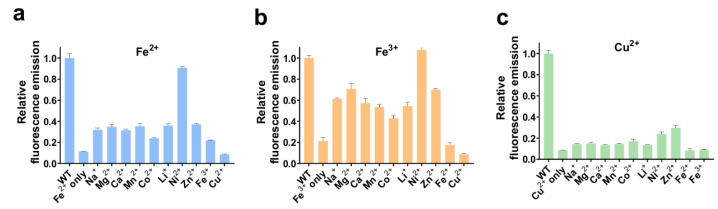
Selectivity. A mixture of (**a**) Fe^2+^, (**b**) Fe^3+^, or (**c**) Cu^2+^ (2 mM) and various metal ions (2 mM) was incubated with tKeima solutions (4 µM). For the control reaction, a solution containing only Fe^2+^, Fe^3+^, or Cu^2+^ without interference metals was used. WT indicates a solution containing only tKeima. Data represent the means ± standard deviations of three replicates. WT, wild type.

Selectivity toward the target ion is a critical criterion for evaluating the performance of FP-based metal biosensors because cross-reactivity with other metal ions may interfere with signal reliability and reduce the sensor’s utility in complex environments [31]. To evaluate the selectivity of tKeima toward Fe^2+^, Fe^3+^, and Cu^2+^, fluorescence quenching assays were conducted in the presence of each metal ion (2 mM) with or without equimolar concentrations of potential interfering metal ions, including Na^+^, Mg^2+^, Ca^2+^, Mn^2+^, Co^2+^, Li^+^, Ni^2+^, and Zn^2+^ (Figure 5). The interfering ions used for the initial screening test (Figure 1) were chosen for the selectivity assays as a standard panel commonly used in fluorescent protein–based metal-sensing systems [2,32]. For Fe^2+^ and Fe^3+^, most tested ions, except for Ni^2+^, caused modest interference, showing about twice the fluorescence emission relative to that of the control without interference (i.e., Fe^2+^ only or Fe^3+^ only). However, both ions exhibited pronounced susceptibility to Ni^2+^, with quenching retention dropping to approximately 90% of that of the control, indicating a significant reduction in selectivity under Ni^2+^ interference. In contrast, Cu^2+^ relatively maintained its quenching response across all tested metal ions, demonstrating robust selectivity, although a small extent of interference by Ni^2+^ and Zn^2+^, exhibiting approximately 20% higher fluorescence emission, was observed. These results highlight that while tKeima exhibits high selectivity for Fe^2+^ and Fe^3+^ against most biologically relevant metal ions, Ni^2+^ represents a notable exception, whereas Cu^2+^ quenching is only slightly affected by the presence of competing ions. Compared with DendFP, which shows robust resistance to interference from other ions but limited discrimination among Fe^2+^, Fe^3+^, and Cu^2+^, tKeima exhibits lower selectivity against interfering ions [14]. However, tKeima provides enhanced selectivity in distinguishing Fe^2+^, Fe^3+^, and Cu^2+^, the target ions of interest in this study.

### 3.6. LOD and LOQ

The LOD and LOQ are essential parameters in biosensor evaluation, as they determine the minimum analyte levels detectable and accurately measurable by the system [33]. The LOD values were determined to be 396.0 μM for Fe^2+^, 428.6 μM for Fe^3+^, and 475.7 μM for Cu^2+^, while the corresponding LOQ values were 1320.0 μM, 1428.6 μM, and 1586.8 μM, respectively. These results indicate that tKeima exhibits the highest detection sensitivity toward Fe^2+^, followed by Fe^3+^ and Cu^2+^.

### 3.7. Application for Realistic Matrices

**Figure 6:**
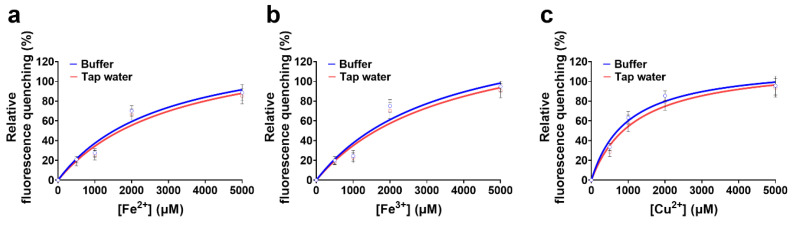
Matrix effects of tap water on tKeima quenching. Relative fluorescence quenching of tKeima in 50 mM Tris-HCl buffer (pH 8.0) and tap water as a function of (**a**) Fe^2+^, (**b**) Fe^3+^, or (**c**) Cu^2+^ concentration (0–5000 μM). tKeima (4 μM) was incubated with the indicated metal concentrations under each matrix condition to assess matrix-derived interference. Data represent the means ± standard deviations of three replicates.

**Figure 7:**
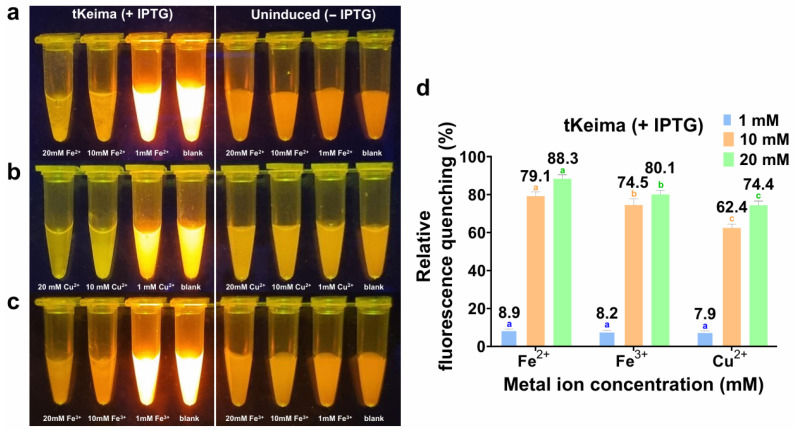
Whole-cell-based fluorescence quenching of tKeima by metal ions. The fluorescence images of tKeima-expressing whole cells (+ IPTG) and uninduced cells (− IPTG), visualized under LED excitation, after incubation with (**a**) Fe^2+^, (**b**) Fe^3+^, or (**c**) Cu^2+^ at 1, 10, and 20 mM; the metal-free condition was used as the blank. (**d**) Quantitative analysis of whole-cell quenching (%) for tKeima (+ IPTG) in response to Fe^2+^, Fe^3+^, and Cu^2+^ at 1, 10, and 20 mM. Different letters above bars indicate significant differences, determined by one-way ANOVA (*p* < 0.05). Data represent the means ± standard deviations of three replicates.

Beyond these analytical parameters, we further examined the behavior of tKeima in realistic matrices, such as tap water and whole cells (Figure 6 and Figure 7); all fits showed a high goodness of fit (*R*^2^ > 0.95). Fe and Cu species were selected as practically relevant targets because they are commonly encountered in complex environmental and biological matrices. First, metal titration experiments in tap water showed that although the baseline fluorescence intensity decreased by approximately 10–20% relative to buffer, the fitted *K*_d_ values for Fe^2+^, Fe^3+^, and Cu^2+^ remained within a similar range across the two matrices (Figure 6). These results indicate that matrix-derived components mainly affect fluorescence brightness rather than metal-binding equilibrium, and that tKeima retains its characteristic quenching behavior even in environmental water analysis. Standard deviations were all within 10%, indicating high reproducibility (Supplementary Figure S3). The pH-dependent fluorescence measurements from previous experiments (Supplementary Figure S1) further confirmed that tKeima remains spectrally stable within a slightly acidic to basic window (approximately pH 5–9), which encompasses the pH range typically found in environmental water samples. For practical environmental water analysis, immobilization of the protein sensor onto solid supports would be required to improve signal stability, mitigate matrix or pH interference effects, enable washing steps, and facilitate repeated or continuous measurements [34].

To assess whether metal-induced quenching is retained in a cellular environment, tKeima was evaluated in a whole-cell format (Figure 7). Uninduced cells (− IPTG) were used as the control to measure cellular autofluorescence, and the concentrations of induced (+ IPTG) and uninduced (− IPTG) cells were matched to minimize cell-density-dependent scattering and background variation. In whole-cell suspensions, Fe^2+^ caused 8.9%, 79.1%, and 88.3% quenching at 1, 10, and 20 mM, respectively. Fe^3+^ caused 8.2%, 74.5%, and 80.1% quenching, and Cu^2+^ caused 7.9%, 62.4%, and 74.4% quenching at the same concentrations. In contrast, although low-level leaky expression was observed at approximately 3000 arbitrary units (a.u.), the uninduced (− IPTG) cells remained stable with no apparent quenching across all metal concentrations, and induced cells without metal ions displayed much higher fluorescence intensity. Standard deviations were all within 10%, indicating high reproducibility (Supplementary Figure S3). These results indicate that the metal-responsive quenching behavior of tKeima is clearly detectable in a whole-cell matrix, despite the presence of cellular constraints and background interference. These further imply that cellular uptake and delivery of metals to tKeima localized within the cytosol were efficiently achieved, as confirmed by SDS-PAGE (Supplementary Figure S4). Taken together, tKeima shows potential applications in cellular and environmental monitoring. To extend its application to real environmental monitoring, further validation using real contaminated matrices, such as industrial wastewater and polluted soil extracts, by analyzing matrix interference and spike recovery will be required.

Electrochemical and aptamer-based sensors typically achieve nM–pM detection limits (Table 1) [35]. For example, a stripping-voltammetry electrochemical sensor achieved an LOD of 0.0048 nM with a linear range of 0.0007–1.5000 μM for Cu^2+^ sensing, showing a relative error of within ±5% against interfering ions [36]. However, they require specialized instrumentation and are highly sensitive to ionic strength, pH, and matrix fouling [35,36]. Sensors often operate in the μM–mM range, with an LOD of 0.245 μM and a linear range of 0.39–78.68 μM in genetic-circuit *E. coli* whole-cell systems [37]. However, whole-cell sensors often suffer not only from poor selectivity owing to non-specific cellular responses to metal ions but also from practical issues related to viability and regulation [38]. Regarding reversibility, electrochemical platforms show high reversibility due to reversible stripping processes [39], whereas aptamer-based sensors exhibit limited signal recovery despite reversible binding [40]. Whole-cell sensors have limited reusability because cellular dynamics prevent a complete signal reset [41]. Overall, despite its moderate sensitivity, we propose that tKeima’s performance can be positioned within the broader landscape of metal-ion biosensors, considering its high selectivity (particularly for Cu^2+^) and partial reversibility. Together with its large Stokes shift and spectral robustness, tKeima could be particularly advantageous for rapid optical screening of relatively high metal ion concentrations under complex or heterogeneous conditions. This is directly applicable to first-line screening of Cu/Fe in high-load industrial wastewaters [42] and in protein- or peptide-rich samples where metal coordination to amino-acid moieties, such as histidine and cysteine, can modulate the apparent metal availability under complex matrices [43]. Under these conditions, sensors with moderate metal-binding affinity may provide more stable responses within the dynamic metal–ligand exchange regime.

### 3.8. Structural Analysis of tKeima for Metal Ion Binding

**Figure 8:**
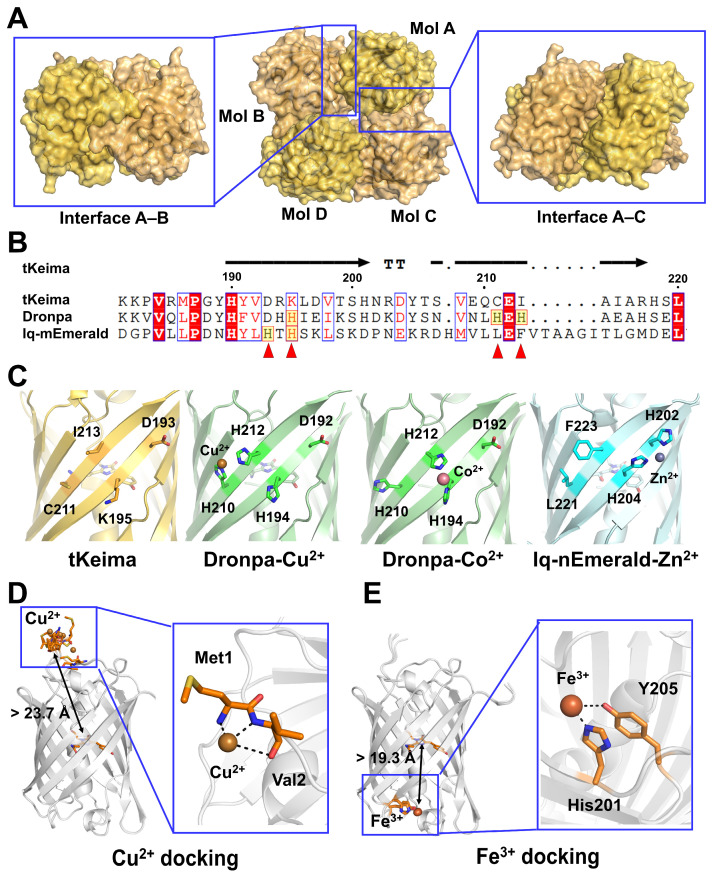
Structural analysis of tKeima for quenchable metal ion binding. (**A**) Tetrameric structure of tKeima (PDB code: 8XC6). The quenchable metal ion-accessible surface of tKeima is indicated by red arrows. (**B**) Partial amino acid sequence alignment of tKeima (UniProt: Q1JV72) with the quenchable metal ion binding sites of iq-mEmerald (P42212) and Dronpa (UniProt: Q5TLG6). The binding sites in iq-mEmerald and Dronpa are indicated by red arrows. (**C**) Structural comparison of the surface of tKeima with the quenchable metal ion binding sites of iq-mEmerald (PDB code: 4KW4) and Dronpa (5HZT for Cu^2+^, and 5HZS for Co^2+^). Docking of (**D**) Cu^2+^ and (**E**) Fe^3+^ to tKeima using AlphaFold3.

The crystal structure of tKeima (PDB code: 8XC6) revealed a tetrameric assembly with 222 symmetry (Figure 8A). The tetrameric interface of tKeima is stabilized by extensive hydrogen bonds and salt bridges between tKeima molecules [13], indicating that quenchable metal ions cannot access this interface. Structural analysis showed that each tKeima monomer has a solvent-accessible surface area of approximately 10,277 Å^2^, of which 1978 Å^2^ is buried at the interface. This suggests that quenchable metal ions can potentially bind only to approximately 80% of the total surface area of tKeima that is solvent-exposed. 

To date, the crystal structures of the FPs iq-mEmerald, Dronpa, and BFPms1 bound to quenchable metal ions have been reported. In iq-mEmerald and Dronpa, the metal ion is commonly coordinated by histidine residues located on the β-barrel surface, but the positions of these histidine molecules and the geometry of metal coordination differ between the two proteins. In iq-mEmerald, the NE atoms of His202 and His204 interact with Zn^2+^, which is located 14.4 Å away from the chromophore (measured from the center of the imidazole ring). In Dronpa, Cu^2+^ interacts with His210 and His212, while Co^2+^ interacts with His194 and His212. The distances of Cu^2+^ and Co^2+^ bound to Dronpa from the center of the imidazole ring to the chromophore are 14.9 Å and 14.4 Å, respectively. Sequence alignment and structural analysis further demonstrated that the quenchable metal ion binding site of iq-mEmerald and Dronpa differs from that of tKeima (Figure 8B–D). The positions of the quenchable metal ion-binding histidine residues in iq-mEmerald and Dronpa correspond to Asp, Lys, Cys, and Ile residues in tKeima, indicating that the quenchable metal ion-binding site of tKeima differs from those of iq-mEmerald and Dronpa (Figure 8B,C). Meanwhile, in BFPms1, divalent metal ions were bound to the chromophore through an engineered hole in the β-barrel structure [44]. As the surface of tKeima lacks such a hole, the mechanism of fluorescence quenching by metal ions in tKeima must differ from that in BFPms1. 

AF3 is an AI-based protein prediction tool that demonstrates higher accuracy for protein–ligand interactions compared with state-of-the-art docking methods [20]. Recently, quenchable metal ion docking to the iq-mEmerald and Dronpa FPs was analyzed using AF3 and compared with their experimental results [45]. These results showed that although the coordination of the docked metal ions differed from the experimental results, some AI-predicted models positioned divalent metal ions near histidine residues involved in metal ion binding in the experimental structures of iq-mEmerald and Dronpa. To investigate the divalent metal ion-induced quenching mechanism of tKeima, Cu^2+^ and Fe^3+^, which are available as ligands in AF3, were docked into the tKeima molecule. For Cu^2+^, the ipTM and pTM scores of the docking models were 0.51–0.59 and 0.91–0.92, respectively, showing that all Cu^2+^ ions were located in the N-terminal region of tKeima (Figure 8D). These docked Cu^2+^ ions interacted with the N or O atoms of the main chain of Met1 and Val2 at distances of 1.73–3.48 Å. For Fe^3+^, the ipTM and pTM scores of the docking models were 0.88–0.90 and 0.92, respectively, showing that all Fe^3+^ ions were located at the bottom of the β-barrel fold of tKeima (Figure 8E). These docked Fe^3+^ ions interacted with the NE2 atom of His201 and the hydroxyl group of Tyr205 at distances of 2.14–2.20 and 3.33–3.65 Å, respectively. Spectral analysis showed that the distance between the chromophore and Cu^2+^, corresponding to the R_0_ FRET values, was in the range of 7–20 Å, but the distance between the quenchable metal ion binding site and the chromophore is critical for the fluorescence quenching effect [46]. The AF3-generated model structure does not include the chromophore as a post-translational modification. Accordingly, when the docking results were compared with the experimental chromophore structure of tKeima, the distances of the docked Cu^2+^ and Fe^3+^ ions to the chromophore via the imidazole ring in tKeima were 23.7–29.2 Å and 19.3–19.7 Å, respectively (Figure 8D,E). These results indicate that the fluorescence quenching effect of the docked Cu^2+^ and Fe^3+^ metal ion binding sites on tKeima may be negligible or very weak. Another possible mechanism for metal-induced quenching of tKeima is that, instead of direct interaction of metal ions with the protein’s binding site, fluorescence quenching occurs in a distance-dependent manner between the metal ions and the chromophore, as previously shown for Cu^2+^ interaction with ZsYellow. This is consistent with the lower sensitivity of tKeima toward metal ions compared with other FPs, such as DendFP [14] and Dronpa [21], both of which were verified to possess a metal-binding site by crystallographic studies. Since our interpretation of the quenching mechanism is based on spectroscopic analyses and computational docking rather than experimental structural data, the proposed binding sites should be considered provisional. To clarify the quenchable metal ion binding site in tKeima, further crystallographic or small-angle X-ray scattering (SAXS) studies will be required to understand divalent metal ion-induced fluorescence quenching, which may provide valuable insights for engineering tKeima as an enhanced FP-based metal biosensor. To enhance the binding affinity, strategies to strengthen metal interactions are required [47]. Structure-guided mutagenesis of surface residues that shape the electrostatic and hydrogen-bonding environment around the chromophore (e.g., Glu145, Asp158, Gln210, Glu212, or Arg92), or the introduction of histidine-rich or acidic residues that mimic the well-characterized metal-binding motifs of Dronpa or iq-mEmerald, together with buffer optimization (e.g., ionic strength and removal of weak chelators), could increase coordination-driven metal binding [32].

Compared with previous reports on other fluorescent proteins [10,14,21,22], our study provides new insights into metal-induced quenching in terms of both mechanism and application through systematic and quantitative analysis, AF3-based docking, and evaluation in realistic matrices. Notably, this is one of the few studies to elucidate detailed quenching mechanisms by integrating multiple model-based analyses. Furthermore, it is the first to combine mechanistic characterization with practical validation in complex environments. Together, these findings establish a comprehensive framework for understanding and applying tKeima as a metal-responsive fluorescent platform.

## 4. Conclusions

Overall, this study provides the first comprehensive characterization of metal-induced fluorescence quenching of the large-Stokes-shift FP tKeima and establishes its potential as a robust and stable fluorescent platform for monitoring metal ion dynamics under complex or physiological conditions. The moderate sensitivity of tKeima, defined by key analytical parameters—LOD values of 396 μM (Fe^2+^), 429 μM (Fe^3+^), and 458 μM (Cu^2+^); LOQ values of 1320 μM (Fe^2+^), 1429 μM (Fe^3+^), and 1587 μM (Cu^2+^); and *K*_d_ values of 2710.7 ± 178.6 μM (Fe^2+^), 3112.0 ± 176.7 μM (Fe^3+^), and 881.9 ± 76.2 μM (Cu^2+^)—offers a practical benefit for applications involving higher metal ion concentrations. In addition, tKeima’s exceptionally large Stokes shift, strong spectral stability, and partially reversible quenching further highlight its utility as a stable and interference-resistant fluorescent probe. Together, these findings underscore tKeima as a promising and versatile biosensing platform and provide foundational data for the development of next-generation metal-responsive fluorescent sensors.

## Figures and Tables

**Table 1:** Comparison of performance among various sensor platforms.

Platform	LOD Level	Selectivity	Reversibility	Reference
tKeima	396–479 μM	High (Cu^2+^)	Partial (~40% recovery at 500 mM EDTA)	This study
Other fluorescent proteins (DendFP, Dronpa, Zsyellow)	3–7 μM (DendFP)	Variable	Partial (~65% recovery at 500 mM EDTA)	[14,21,22]
Electrochemical	4.8 pM	High (±5% error)	High (Reversible stripping processes)	[36]
Aptamer	1 pM–5 nM	-	Limited (Limited signal recovery)	[35]
Whole-cell	0.4–78.7 μM	Low (Non-specific cellular responses)	Limited	[37]

## Data Availability

The datasets used and/or analyzed during the current study are available from the corresponding author upon reasonable request.

## Supplementary Materials

The following supporting information can be downloaded at: https://www.mdpi.com/article/10.3390/bios16030178/s1, Figure S1: pH effect on tKeima fluorescence quenching; Figure S2: Stern–Volmer analysis of tKeima quenching by Fe^2+^, Fe^3+^, and Cu^2+^ at different temperatures; Figure S3: Long-term fluorescence stability of Cu^2+^—induced quenching in tKeima; Figure S4: SDS-PAGE analysis of recombinant tKeima expressed in *E. coli* and its purification by Ni–NTA affinity chromatography.
