# Supplementary material for: tKeima: A Large-Stokes-Shift Platform for Metal Ion Detection
Source: Biosensors (Basel). 2026 Mar 22;16(3):178. doi: 10.3390/bios16030178 (PMC13023891; doi:10.3390/bios16030178)
Supplement: Supplementary file 1 [file biosensors-16-00178-s001.zip › biosensors-4169468-supplementary.pdf]

Supplementary Information

tKeima: A Large-Stokes-Shift Platform for Metal Ion Detection

Yun Gyo Seo [1], Dan-Gyeong Han [1] and In Jung Kim [2,*]

[1]Division of Applied Life Sciences, College of Agriculture and Life Science, Gyeongsang National University, Jinju 52828, Republic of Korea

[2]Department of Food Science & Technology, College of Agriculture and Life Science, Gyeongsang National University, Jinju 52828, Republic of Korea

[*] Correspondence: ij0308@gnu.ac.kr

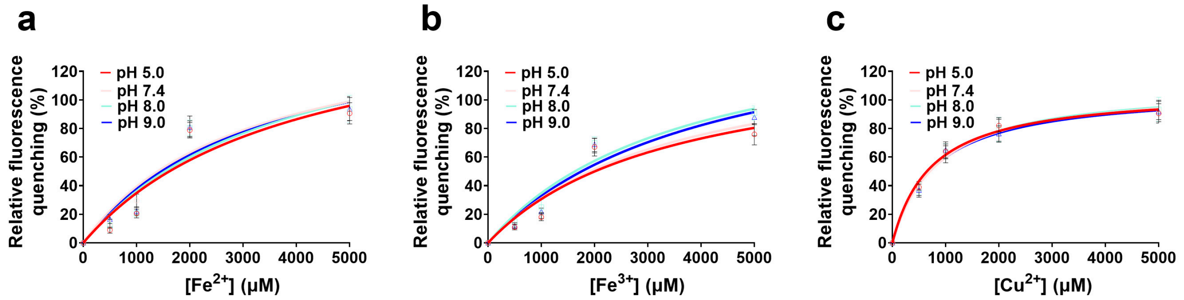

Figure S1. pH effect on tKeima fluorescence quenching. tKeima was incubated with (a) Fe^{2+}, (b) Fe^{3+}, and (c) Cu^{2+} under pH 5.0, 7.4, 8.0, and 9.0 conditions over a concentration of range of 0-5000 μM. Data are expressed as means ± standard deviations from three independent experiments.

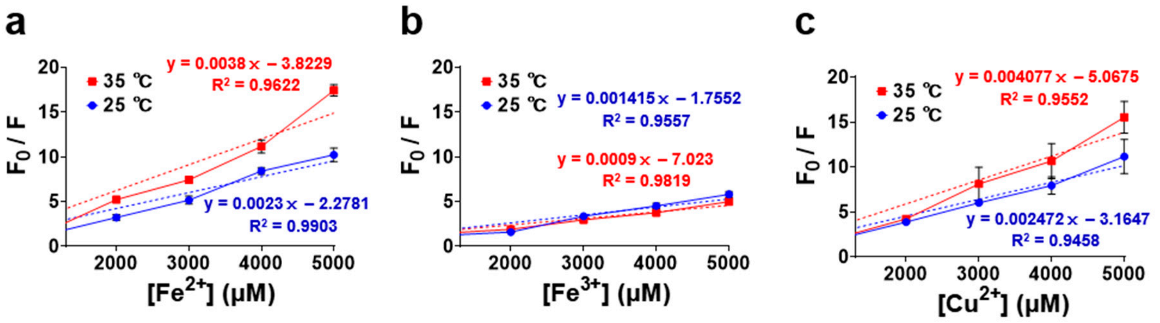

Figure S2. Stern-Volmer analysis of tKeima quenching by Fe^{2+}, Fe^{3+}, and Cu^{2+} at different temperatures.

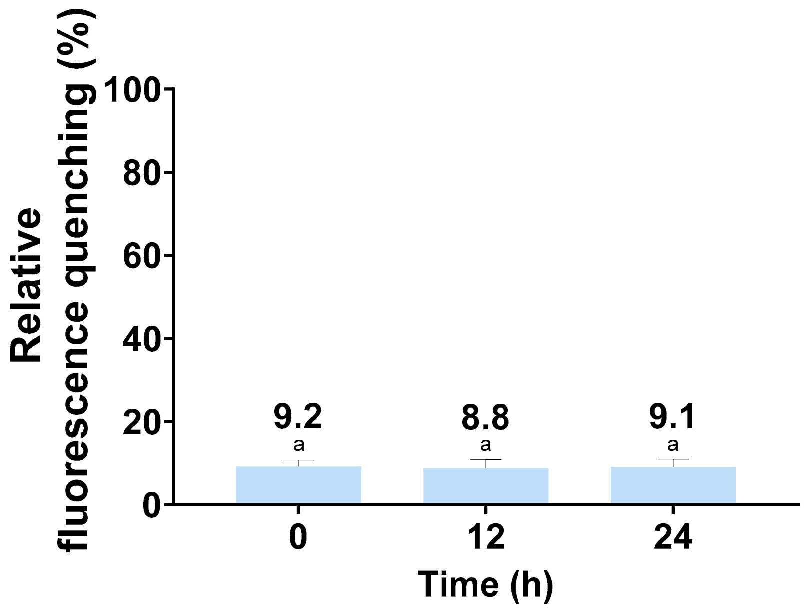

Figure S3. Long-term fluorescence stability of Cu^{2+}- induced quenching in tKeima. tKeima was incubated with 2 mM Cu^{2+} for 5 min, and fluorescence quenching was measured at 0, 12, and 24 h. Data are expressed as means ± standard deviations from three independent experiments. The letters above bar indicates non-statistical difference, determined by one-way ANOVA ($p > 0.05$).

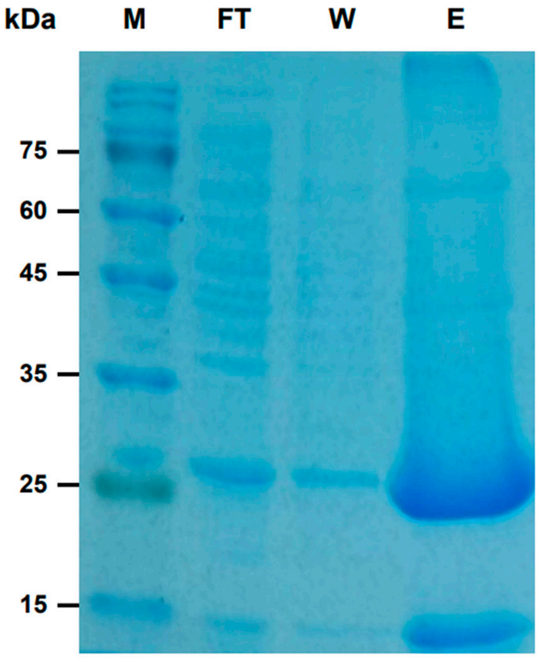

Figure S4. SDS-PAGE analysis of recombinant tKeima expressed in *E. coli* and its purification by Ni–NTA affinity chromatography.

The molecular weight marker is shown in lane M. The flow-through (FT) and wash (W) fractions represent proteins that did not bind or were weakly bound to the Ni–NTA resin, whereas the elution (E) fraction contains the His-tagged tKeima recovered from the resin. A prominent band at approximately ~25 kDa, consistent with the expected molecular weight of tKeima, was enriched in the elution fraction.
